# HSP90β Impedes STUB1‐Induced Ubiquitination of YTHDF2 to Drive Sorafenib Resistance in Hepatocellular Carcinoma

**DOI:** 10.1002/advs.202302025

**Published:** 2023-07-28

**Authors:** Yuning Liao, Yuan Liu, Cuifu Yu, Qiucheng Lei, Ji Cheng, Weiyao Kong, Yuanhui Yu, Xuefen Zhuang, Wenshuang Sun, Shusha Yin, Gengxi Cai, Hongbiao Huang

**Affiliations:** ^1^ Affiliated Cancer Hospital & institute of Guangzhou Medical University Guangzhou 510095 China; ^2^ Guangzhou Municipal and Guangdong Provincial Key Laboratory of Protein Modification and Degradation School of Basic Medical Sciences Guangzhou Medical University Guangzhou 511436 China; ^3^ Shenshan Medical Center Memorial Hospital of Sun Yat‐sen University Shanwei 516600 China; ^4^ Department of Hepatopancreatic Surgery The First People's Hospital of Foshan Foshan 528000 China; ^5^ KingMed School of Laboratory Medicine Guangzhou Medical University Guangzhou 511436 China; ^6^ Department of Breast Surgery The First People's Hospital of Foshan Foshan 528000 China

**Keywords:** hepatocellular carcinoma, HSP90β, STUB1, ubiquitination, YTHDF2

## Abstract

YTH domain family 2 (YTHDF2) is the first identified N6‐methyladenosine (m^6^A) reader that regulates the status of mRNA. It has been reported that overexpressed YTHDF2 promotes carcinogenesis; yet, its role in hepatocellular carcinoma (HCC) is elusive. Herein, it is demonstrated that YTHDF2 is upregulated and can predict poor outcomes in HCC. Decreased ubiquitination levels of YTHDF2 contribute to the upregulation of YTHDF2. Furthermore, heat shock protein 90 beta (HSP90β) and STIP1 homology and U‐box‐containing protein 1 (STUB1) physically interact with YTHDF2 in the cytoplasm. Mechanically, the large and small middle domain of HSP90β is required for its interaction with STUB1 and YTHDF2. HSP90β inhibits the STUB1‐induced degradation of YTHDF2 to elevate the expression of YTHDF2 and to further boost the proliferation and sorafenib resistance of HCC. Moreover, HSP90β and YTHDF2 are upregulated, while STUB1 is downregulated in HCC tissues. The expression of HSP90β is positively correlated with the YTHDF2 protein level, whereas the expression of STUB1 is negatively correlated with the protein levels of YTHDF2 and HSP90β. These findings deepen the understanding of how YTHDF2 is regulated to drive HCC progression and provide potential targets for treating HCC.

## Graphical abstract

This study explores the ubiquitination of the N6‐methyladenosine (m^6^A) reader, YTH domain family 2 (YTHDF2). Heat shock protein 90 beta (HSP90β) promotes the progression of hepatocellular carcinoma (HCC) by suppressing STIP1 homology and U‐box‐containing protein 1 (STUB1)‐induced YTHDF2 ubiquitination, which may inaugurate a novel intervention strategy for the treatment of HCC.

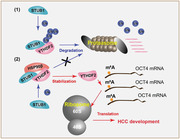

## 1 Introduction

Hepatocellular carcinoma (HCC) is a challenging disease with high incidence and fatality,^[^ ^1^ ^]^ and extremely poor survival (less than 6%), strongly associated with late tumor diagnosis.^[^ ^1^ ^]^ Additionally, due to the high heterogeneity,^[^ ^2^ ^]^ patients with HCC can hardly benefit from a specific therapy. Although hundreds of clinical trials assessing the effect of chemotherapy, radiotherapy, surgical treatment, targeted therapy, or combination therapy to treat HCC have been performed over the past decades, the survival of HCC patients remains low.^[^ ^1^, ^3^ ^]^ Therefore, developing key therapeutic targets for the effective treatment of HCC is urgent for current medical studies.

As the core of maintaining protein homeostasis and cellular functions, the ubiquitin‐proteasome system (UPS) controls the elimination of most proteins and participates in biological reactions from diverse levels, such as stress response, DNA damage response, and cell proliferation.^[^ ^4^ ^]^ Alteration in UPS is related to many diseases, including various cancers.^[^ ^5^ ^]^ Ubiquitination is a cascade reaction that requires E1, E2, and E3. E3 ligases determine substrate specificity and mediate the ubiquitination of certain oncoproteins, and thus have been proposed as attractive classes of anticancer targets.^[^ ^5^, ^6^ ^]^

Epigenetic modifications are critical in the pathogenesis of many kinds of tumors. Over the years, post‐transcriptional modification has attracted extensive attention in biomedical research. For example, N6‐methyladenosine (m^6^A) methylation, a prevalent mRNA modification in eukaryotic cells, controls the status of mRNA, including RNA processing, translocation, stability, and translation, thereby regulating multiple biological processes.^[^ ^7^ ^]^ Dysregulation of m^6^A methylation is involved in the occurrence and progression of various diseases.^[^ ^7^, ^8^ ^]^ m^6^A methylation is installed by methyltransferases (termed “writers”), such as METTL3/14, WTAP, etc.,^[^ ^8c^ ^]^ which are recognized by m^6^A‐binding proteins (termed “readers”), such as YTHDC1/2, YTHDF1/2/3, etc.^[^ ^8c^ ^]^ Like many other epigenetic modifications, m^6^A methylation is a reversible process. Demethylation is mediated by some specific demethylases (termed “erasers”), such as FTO, ALKBH3/5, etc.^[^ ^8c^ ^]^ YTHDF2, the first identified m^6^A‐binding protein, is upregulated in certain malignant tumors, including gastric cancer, colorectal cancer, etc., and can promote their development.^[^ ^8a^ ^]^ Functionally, YTHDF2 controls protein expressions by regulating the translation and stability of specific mRNAs.^[^ ^8^, ^9^ ^]^ However, the role and modification of YTHDF2 in HCC are still not fully understood.

This study showed that the ubiquitination level of the m^6^A reader YTHDF2 is significantly decreased in HCC. Mechanically, the heat shock protein 90 beta (HSP90β) interacts with YTHDF2 and STIP1 homology and U‐box‐containing protein 1 (STUB1), a well‐characterized E3 ligase, in the cytoplasm with its large and small middle domain. STUB1 triggers ubiquitination and degradation of YTHDF2 via the 26S proteasome, whereas HSP90β blocks this biological process. Consequently, HSP90β boosts the growth and sorafenib insensitivity via deubiquitination and stabilization of YTHDF2. Moreover, our clinical observations showed that the expression of HSP90β or STUB1 is correlated with the protein expression of YTHDF2. In summary, this study furthers the understanding of the regulatory network of YTHDF2 in HCC progression.

## 2 Results

### 2.1 The Ubiquitination Level of YTHDF2 is Downregulated in HCC

**Figure 1:**
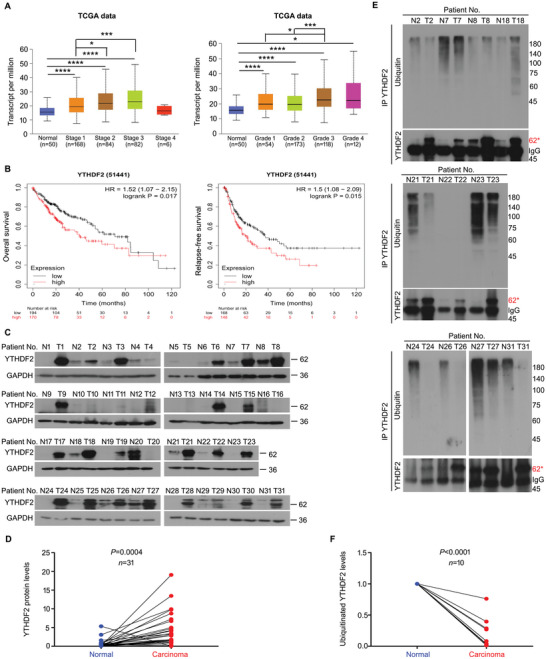
The ubiquitination level of YTHDF2 is downregulated and predicts poor outcomes in HCC. A) Analysis of YTHDF2 mRNA expression in HCC tissues based on cancer stages and tumor grades by analyzing the TCGA and UALCAN databases. *p < 0.05, ^***^ *p* < 0.001, ^****^ *p* < 0.0001. B) Kaplan–Meier curves from HCC patients expressing low and high YTHDF2 from the tissue microarray. Overall survival and relapse‐free survival data are shown. C) YTHDF2 in lysates from the fresh HCC and adjacent normal tissues analyzed by Western blot. GAPDH was used as an internal control. D) Quantification of YTHDF2 in (C). Data were analyzed with paired *t*‐tests. E) Co‐IP/Western blot assays in lysates from the fresh HCC tissues and adjacent normal tissues were performed to determine the levels of ubiquitinated‐YTHDF2 using YTHDF2 antibodies. F) Quantification of ubiquitinated‐YTHDF2 levels in (E). Ubiquitinated‐YTHDF2 was calculated with (ubiquitin density)/(YTHDF2 density) from the Co‐IP/Western blot assays. Data were analyzed with paired *t*‐tests.

To explore whether YTHDF2 is critical for HCC progression, the mRNA expression of YTHDF2 in various stages/grades of HCC was analyzed using the public TCGA database via UALCAN website. YTHDF2 was notably increased in stage 1–3 and grade 1–4; yet, HCC tissues with higher grades showed higher expression of YTHDF2 (**Figure** **1**A). The relationship between YTHDF2 expression and the survival of patients with HCC was further analyzed using the public TCGA database via Kaplan–Meier curves website. We found that upregulation of YTHDF2 was associated with poor outcomes, including overall survival and relapse‐free survival (Figure 1B). Next, the protein expression of YTHDF2 was determined in HCC samples (*n* = 31). We showed that tumor tissues had higher expression of YTHDF2 versus adjacent normal tissues (Figure 1C,D). We next assessed whether the upregulation of YTHDF2 protein expression might result from abnormal ubiquitination of YTHDF2. Co‐immunoprecipitation (co‐IP) analysis was performed in 11 pairs of tumor or adjacent normal tissues among the samples with higher protein expression of YTHDF2. Ubiquitination level of YTHDF2 was determined by the ratio of ubiquitin density/YTHDF2 density in the co‐IP results. The case N22/T22 was finally excluded because the ubiquitination level of YTHDF2 was undetectable in these paired samples. As shown, the ubiquitination level of YTHDF2 was notably decreased in HCC tissues compared with adjacent normal tissues (Figure 1E,F). Together, these findings demonstrate that a deficiency of ubiquitination level contributes to the overexpression of YTHDF2 and drives the malignant progression of HCC.

### 2.2 YTHDF2 Interacts with HSP90β and STUB1

**Figure 2:**
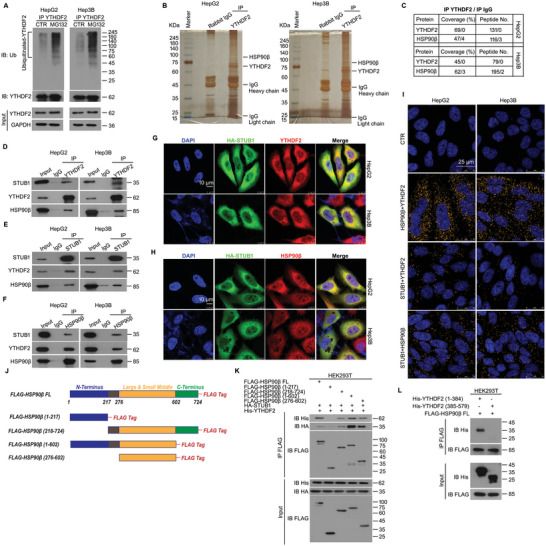
YTHDF2 physically interacts with HSP90β and STUB1 in HCC. A) Co‐IP/Western blot assays were performed using YTHDF2 antibodies in lysates from HCC cells treated with MG132 (10 × 10^−6^ m) for 8 h, subjected to the immunoblotting for ubiquitin (Ub) and YTHDF2. B) Co‐IP assay was performed in HepG2 and Hep3B cell lysates using YTHDF2 or IgG control antibodies. The Co‐IP products were subjected to Sodium dodecyl sulfate‐polyacrylamide gel electrophoresis (SDS‐PAGE) separation, silver staining, and biological mass spectrometry (LC‐MS/MS analysis). C) The peptide numbers and coverage of YTHDF2 and HSP90β from the LC‐MS/MS analysis. D–F) Co‐IP assay was performed in HepG2 and Hep3B cell lysates using YTHDF2, STUB1, HSP90β, or IgG control antibodies, followed by immunoblotting for YTHDF2, STUB1, and HSP90β. G,H) The HA‐labeled STUB1 plasmids were transfected in HepG2 and Hep3B for 48 h. IF assay/confocal microscopy was further performed to observe the subcellular location of YTHDF2, STUB1, and HSP90β. Scale bar, 10 µm. I) PLA assay was performed in HepG2 and Hep3B cells using STUB1, HSP90β, and YTHDF2 antibodies. The orange point represents positive interaction. Scale bar, 25 µm. J) The full length and diverse truncated mutants of HSP90β with FLAG‐tag were constructed. Linear models were shown. K) Diverse truncated mutants of HSP90β were transfected in HEK293T cells with HA‐STUB1 and 6×His‐YTHDF2 plasmids for 48 h. Co‐IP assay was performed using HA antibodies, followed by immunoblotting for FLAG and HA. L) Truncated mutants of 6×His‐YTHDF2 were transfected in HEK293T cells with FLAG‐HSP90β plasmids for 48 h. Co‐IP assay was performed using FLAG antibodies, followed by immunoblotting for FLAG and His.

It has been observed that YTHDF2 can be modified by ubiquitin in HCC samples. Thus, we subsequently examined whether the proteasome may degrade YTHDF2. Our co‐IP results showed that the ubiquitination level of YTHDF2 was notably upregulated post the short exposure of MG132, a potent proteasome inhibitor, in HepG2 and Hep3B cells (**Figure** **2**A), indicating that the canonical ubiquitin‐proteasome pathway degrades YTHDF2. We previously reported that heat‐shock proteins (HSPs/chaperones) have a critical role in controlling the degradation of specific proteins.^[^ ^10^, ^11^ ^]^ In this study, we further investigated whether HSPs may regulate the ubiquitination of YTHDF2 and lead to its abnormal expression. Co‐IP assay combined with liquid chromatography with tandem mass spectrometry (LC‐MS/MS) analysis showed that HSP90β emerged as the most important YTHDF2‐interacting HSPs in HCC cells (Figure 2B,C and Figure S1, Supporting Information). As reported previously,^[^ ^12^ ^]^ STUB1 is an HSP70/90‐interacting E3 ligase. Thus, we performed co‐IP and Western blot to detect their protein interactions using anti‐YTHDF2, anti‐STUB1, and anti‐HSP90β, respectively. Our results showed that YTHDF2, STUB1, and HSP90β can interact with each other (Figure 2D–F). Next, exogenous immunofluorescence (IF), endogenous IF, proximity ligation, and confocal microscopy assays were performed in HCC cells to further clarify the subcellular location of their interactions. These results consistently showed that their interactions were mainly localized in the cytoplasm in HCC cells (Figure 2G–I and Figure S2A, Supporting Information). Thus, we further aimed to investigate whether there is a specific binding domain of HSP90β to STUB1. Truncated mutants and full‐length of HSP90β were engineered in plasmids with FLAG‐tag in their C‐terminals (Figure 2J). These plasmids were transfected with HA‐STUB1, respectively, in HEK293T cells. Co‐IP results showed that the large and small middle domain (276‐602 aa) of HSP90β was critical to its binding to STUB1 and YTHDF2 (Figure 2K). In addition, we found that the N‐terminus (1‐384 aa) of YTHDF2 is required for its binding to HSP90β (Figure 2L). The co‐IP results in HepG2 cells were also consistent with the findings in HEK293T cells (Figure S2B, Supporting Information). Thus, the above results indicated that HSP90β interacts with the N‐terminus of YTHDF2 (1‐384 aa) and STUB1 through its large and small middle domains in the cytoplasm.

### 2.3 HSP90β and STUB1 Regulate the Stability of YTHDF2

**Figure 3:**
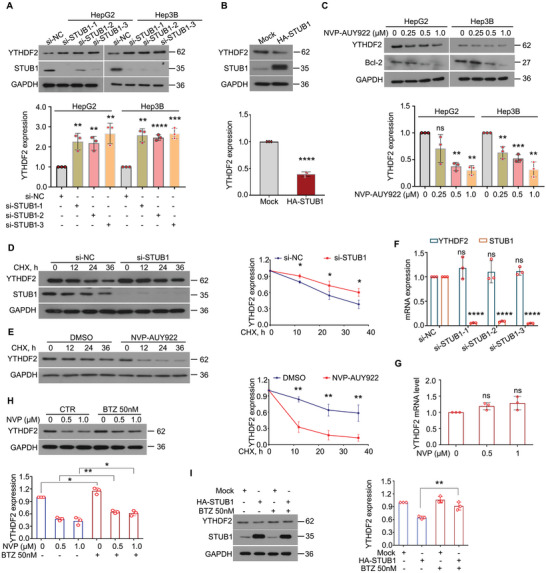
Protein level of YTHDF2 is regulated by HSP90β and STUB1 in HCC. A) Western blot assay for YTHDF2 and STUB1 in HepG2 and Hep3B cells exposed to STUB1 siRNAs or control siRNAs for 72 h. Quantification was shown below the images. B) Western blot assay for YTHDF2 and STUB1 in HepG2 cells exposed to HA‐STUB1 or control plasmids for 48 h. Quantification was shown below the images. C) Western blot assay for YTHDF2 in HepG2 and Hep3B cells exposed to NVP‐AUY922 (NVP) for 48 h. Quantification was shown below the images. D) Western blot assay for YTHDF2 and STUB1 in HepG2 cells exposed to STUB1 or control siRNAs for 48 h, followed by cycloheximide treatment (CHX, 100 µg mL^−1^) for 12, 24, and 36 h. Quantification was shown on the right. E) Western blot assay for YTHDF2 was performed in HepG2 cells exposed to NVP‐AUY922 (0.5 × 10^−6^ m) for 24 h, followed by the treatment of CHX for 12, 24, and 36 h. Quantification was shown on the right. F) RT‐qPCR assays for YTHDF2 and STUB1 were performed in HepG2 cells exposed to STUB1 siRNAs or control siRNAs for 36 h. G) RT‐qPCR assays for YTHDF2 were performed in HepG2 cells exposed to NVP for 12 h. H) Western blot assay for YTHDF2 in HepG2 cells exposed to NVP for 24 h, followed by bortezomib (BTZ) treatment for 24 h. Quantification was shown on the lower side. I) Western blot assay for YTHDF2 and STUB1 in HepG2 cells exposed to HA‐STUB1 or control plasmids for 24 h, followed by the treatment of bortezomib (BTZ) for 24 h. Quantification was shown on the lower side. *p < 0.05, ^**^ *p* < 0.01, ^***^ *p* < 0.001, ^****^ *p* < 0.0001, ns represents not significant.

We further determined the role of STUB1 or HSP90β in YTHDF2 expression. As shown by Western blot, knockdown (KD) of STUB1 upregulated the protein level of YTHDF2 (**Figure** **3**A), whereas the overexpression reversed this process (Figure 3B). Additionally, inhibition of HSP90β with NVP‐AUY922 or *HSP90β*‐KD resulted in the downregulation of YTHDF2 in HCC cells (Figure 3C and Figure S3, Supporting Information). According to a previous report,^[^ ^13^ ^]^ Bcl‐2 was used as a marker to indicate the effect of NVP‐AUY922 in this study. Next, our CHX‐tracking analysis revealed that *STUB1*‐KD significantly postponed the rate of YTHDF2 degradation (Figure 3D), whereas the inhibition of HSP90β notably accelerated the degradation of YTHDF2 (Figure 3E). Meanwhile, neither *STUB1*‐KD nor HSP90β inhibition altered the mRNA level of YTHDF2 (Figure 3F,G). Furthermore, the downregulation of YTHDF2 caused by HSP90β inhibition or STUB1 overexpression was significantly reversed by bortezomib, a specific proteasome inhibitor (Figure 3H,I). Together, these findings indicated that STUB1 reduces the protein stability of YTHDF2, whereas HSP90β increases the protein stability of YTHDF2 in HCC.

### 2.4 HSP90β Inhibits the STUB1‐Induced Ubiquitination of YTHDF2

**Figure 4:**
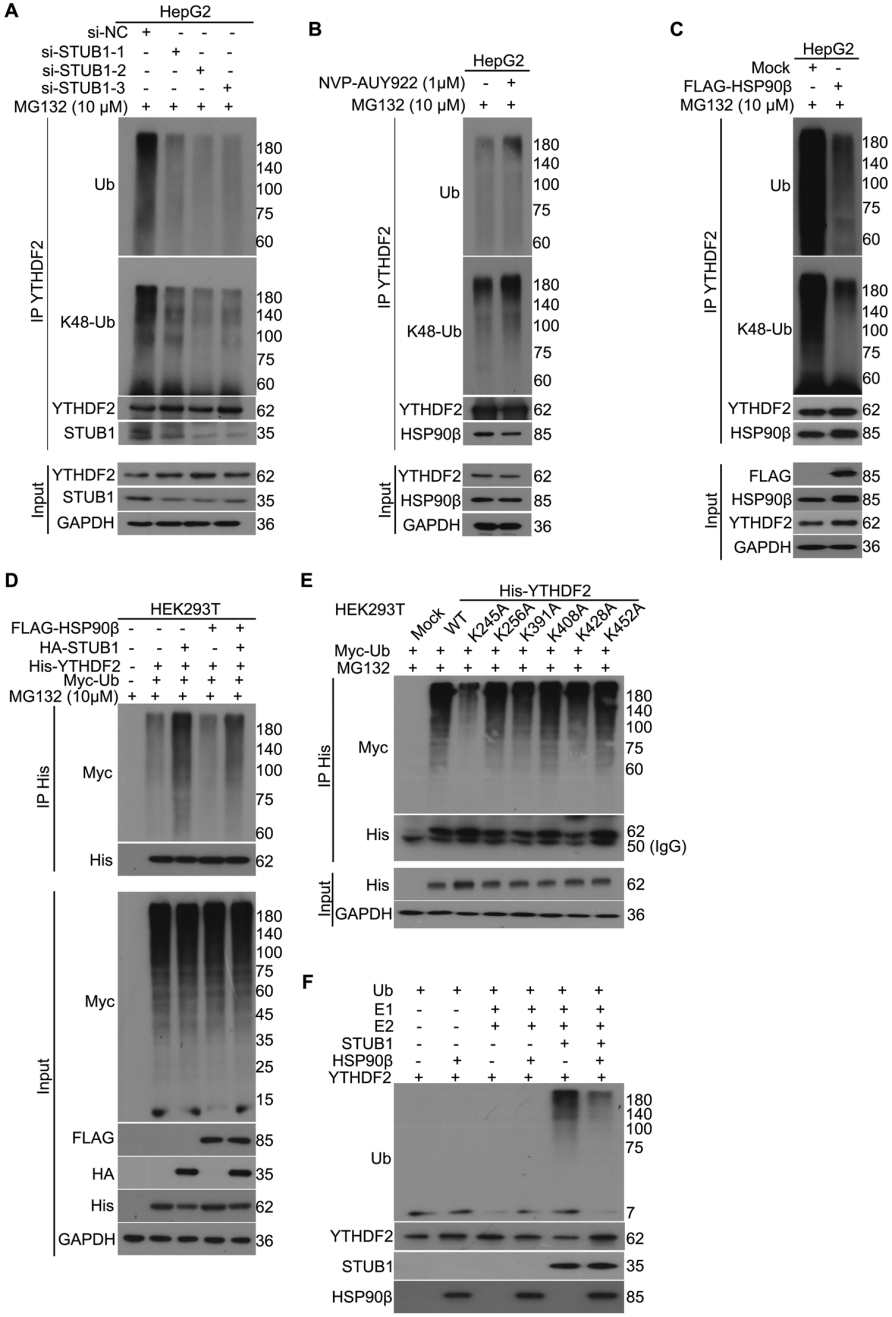
Ubiquitination level of YTHDF2 is controlled by the balance of HSP90β and STUB1. A) Co‐IP assays were performed using YTHDF2 antibodies in lysates from HepG2 cells exposed to STUB1 siRNAs or control siRNAs for 48 h, subjected to the immunoblotting for ubiquitin (Ub), K48‐linked ubiquitin (K48‐Ub), STUB1, and YTHDF2. MG132 was used to treat the cells for 8 h before harvest. B) Co‐IP assays were performed using YTHDF2 antibodies in lysates from HepG2 cells exposed to NVP or vehicle control in the presence of MG132 for 8 h, subjected to the immunoblotting for Ub, K48‐Ub, and YTHDF2. C) Co‐IP assays were performed using YTHDF2 antibodies in lysates from HepG2 cells transfected with FLAG‐HSP90β or control plasmids, subjected to the immunoblotting for Ub, K48‐Ub, YTHDF2, HSP90β, and STUB1. MG132 was used to treat the cells for 8 h before harvest. D) Co‐IP assays were performed using His‐tag antibodies in lysates from HEK293T cells transfected with 6×His‐YTHDF2 and Myc‐Ub plasmids, with or without the transfection of FLAG‐HSP90β or HA‐STUB1 plasmids for 48 h, subjected to the immunoblotting for Myc‐tag and His‐tag. MG132 was used to treat the cells for 8 h before harvest. E) Co‐IP assays were performed using His‐tag antibodies in lysates from HEK293T cells transfected with various Lys‐mutant types of 6×His‐YTHDF2 and Myc‐Ub plasmids for 48 h, subjected to the immunoblotting for Myc‐tag and His‐tag. MG132 was used to treat the cells for 8 h before harvest. F) In vitro ubiquitination assay was performed using the ubiquitinylation kit and specific purified proteins as indicated.

To further determine whether HSP90β and STUB1 may alter the ubiquitination of YTHDF2, co‐IP assays were performed in HepG2 cells treated with si‐STUB1, NVP‐AUY922, or FLAG‐HSP90β plasmids. Our co‐IP analysis showed that the K48‐linked ubiquitination and pan‐ubiquitination levels of YTHDF2 were notably downregulated by *STUB1*‐KD in HCC cells, while they were upregulated by HSP90β inhibition (**Figure** **4**A,B). In addition, overexpression of HSP90β reduced the levels of K48‐linked ubiquitination and pan‐ubiquitination of YTHDF2, and decreased the interaction between STUB1 and YTHDF2 (Figure 4C). To explore whether the regulation of the YTHDF2 by HSP90β is really mediated by STUB1, co‐IP assays were performed in HEK293T cells transfected with Myc‐Ub, 6×His‐YTHDF2, HA‐STUB1, or FLAG‐HSP90β plasmids. The results showed that overexpression of STUB1 notably increased the ubiquitination of YTHDF2, while further overexpression of HSP90β reduced the level of STUB1‐induced ubiquitination of YTHDF2 (Figure 4D).

Ubiquitination mostly occurs at Lys residue. To investigate the ubiquitination site on YTHDF2, six plasmids containing Lys‐mutant types of YTHDF2 were established according to GPS‐Uber, a website to help ubiquitination site prediction. These plasmids were transfected into HEK293T cells, respectively. Our co‐IP results showed that the ubiquitination level of YTHDF2 (K245A), but not other mutant type of YTHDF2, was downregulated, indicating that K245 is a critical ubiquitination site on YTHDF2 (Figure 4E). Moreover, our in vitro ubiquitination assay showed that STUB1 directly triggered ubiquitination of YTHDF2, whereas HSP90β blocked the STUB1‐induced ubiquitination (Figure 4F). Together, our findings indicated that HSP90β blocks the STUB1‐induced ubiquitination of YTHDF2, thereby maintaining the protein level of YTHDF2 in HCC cells.

### 2.5 HSP90β/STUB1 Regulates the Proliferation of HCC in a YTHDF2‐Dependent Manner

**Figure 5:**
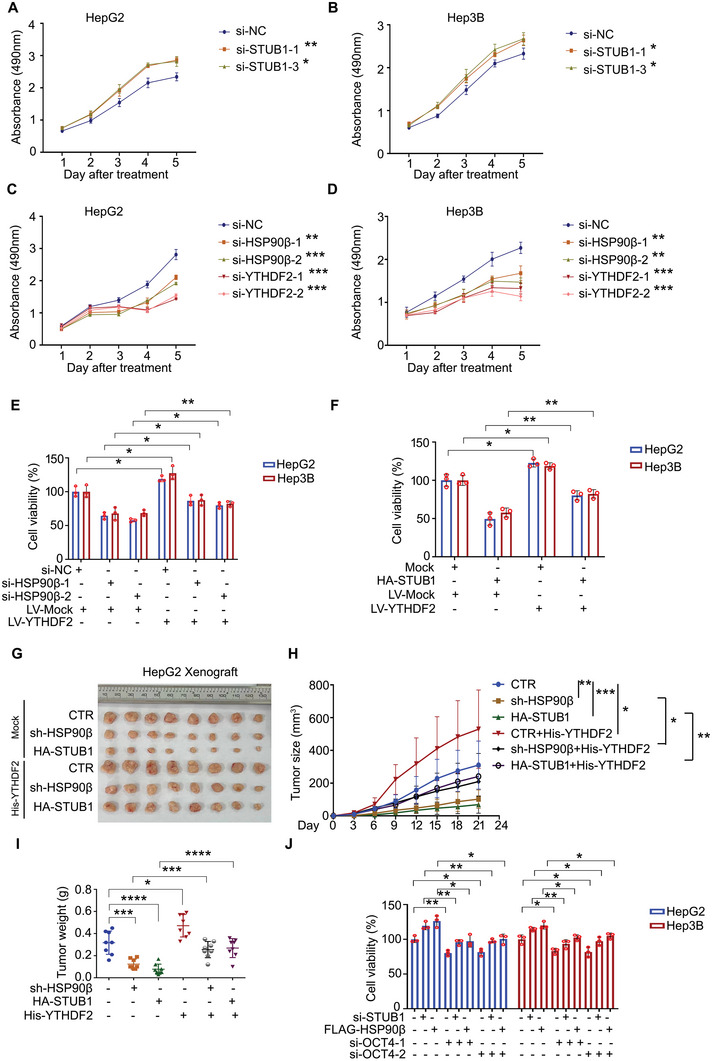
HSP90β and STUB1 regulate the proliferation of HCC in a YTHDF2‐dependent manner. A–D) Cell viability analyses were performed in HepG2 and Hep3B cells treated with STUB1/YTHDF2/HSP90β siRNAs or control siRNAs for 5 days. The OD values were measured every day. E) Cell viability analyses were performed in HepG2 and Hep3B cells stably expressing 6×His‐YTHDF2 or control plasmids, and subjected to the treatment with HSP90β siRNAs or control siRNAs for 72 h. F) Cell viability analyses were performed in HepG2 and Hep3B cells stably expressing 6×His‐YTHDF2 or control plasmids, and subjected to the transfection with HA‐STUB1 or control plasmids for 72 h. G–I) HepG2 cells stably expressing 6×His‐YTHDF2 or control plasmids, with or without HSP90β shRNAs or HA‐STUB1 plasmids, were transplanted on BALB/c nude mice for 3 weeks. Tumor volume was recorded every 3 days. Tumor image, tumor size, and tumor weight were shown. J) Cell viability analyses were performed in HepG2 and Hep3B cells treated with STUB1 siRNAs or FLAG‐HSP90β, with or without the transfection of OCT4 siRNAs for 72 h. *p < 0.05, ^**^ *p* < 0.01, ^***^ *p* < 0.001, ^****^ *p* < 0.0001.

Next, we assessed whether HSP90β, YTHDF2, and STUB1 might be functional in regulating malignant phenotypes of HCC. Cell viability assays were conducted on consecutive 5 days post the treatment of *HSP90β/YTHDF2/STUB1*‐KD to observe cell proliferation. *STUB1*‐KD promoted the proliferation of HCC cells (**Figure** **5**A,B), whereas *HSP90β*‐KD or *YTHDF2*‐KD suppressed HCC proliferation (Figure 5C,D). We further determined whether HSP90β/STUB1 may regulate the proliferation of HCC in a YTHDF2‐dependent manner. Our cell viability assay showed that overexpression of YTHDF2 significantly reversed the growth inhibition induced by the *HSP90β*‐KD or overexpression of STUB1 in HepG2 and Hep3B cells (Figure 5E,F). In addition, in vivo assay showed that overexpression of YTHDF2 rescued the tumor suppression induced by the *HSP90β*‐KD or overexpression of STUB1 in HepG2 xenografts (Figure 5G–I). Next, we aimed to determine which mRNA might be regulated by YTHDF2 to drive HCC progression. It has been reported that OCT4 is a downstream effector for YTHDF2 regulating liver cancer stem cell phenotype via m^6^A RNA methylation. YTHDF2 upregulates the m^6^A level in the 5′‐untranslated region of OCT4 mRNA to elevate the translation and expression of OCT4.^[^ ^14^ ^]^ Thus, we next assessed whether OCT4 mediates the HSP90β/STUB1‐regulated cell proliferation in HCC. The results showed that *OCT4*‐KD significantly reversed the growth promotion induced by the *STUB1*‐KD or overexpression of HSP90β (Figure 5J). Together, these findings illuminate that HSP90β and STUB1 have opposite roles in HCC cells, which is largely associated with their opposite functions in regulating the ubiquitination of YTHDF2.

### 2.6 HSP90β Blockade Restores the Responsiveness of HCC to Targeted Therapy

**Figure 6:**
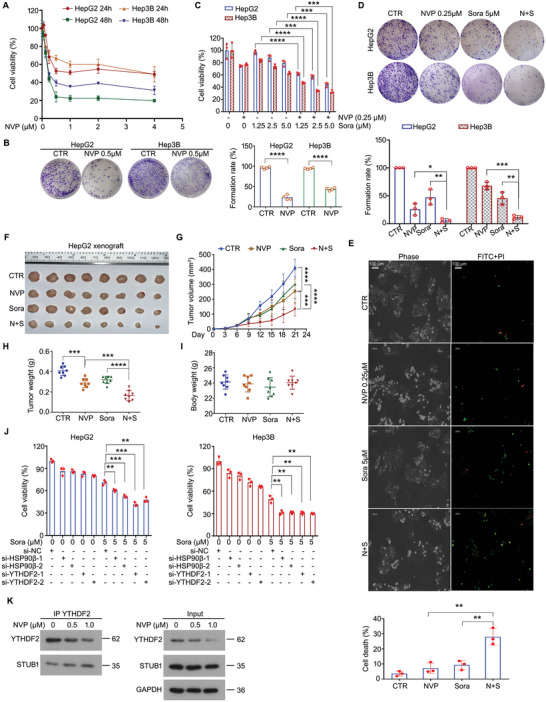
Inhibition of HSP90β sensitizes HCC cells to the treatment of sorafenib. A) Cell viability analyses were performed using MTS assay in HepG2 and Hep3B cells exposed to NVP for 24 and 48 h. B) HepG2 and Hep3B cells were treated with NVP or vehicle for 24 h. Plate colony formation assay was performed post‐treatment. Images were shown on the left, while the quantitative data were shown on the right. C) Cell viability analyses were performed using MTS assay in HepG2 and Hep3B cells exposed to sorafenib with or without NVP for 24 h. D) HepG2 and Hep3B cells were treated with sorafenib with or without NVP for 24 h. Plate colony formation assay was performed post‐treatment. Images were shown on the upper side, while the quantitative data were shown on the lower side. E) Annexin V‐FITC/PI staining assays were performed in HepG2 cells treated with sorafenib with or without NVP for 24 h. Green indicates FITC positive, and red indicates PI positive. Scale bar, 100 µm. Quantification was shown below the images. F) HepG2 xenografts were established and grown in BALB/c nude mice. The mice were divided into four groups and treated with NVP (i.p. 25 mg kg^−1^/2 days), sorafenib (p.o. 20 mg kg^−1^/2 days), the combination of NVP and sorafenib, or vehicle for 3 weeks. Images of the xenografts. G) Tumor volume was recorded every 3 days. The curves of tumor volume. H) Tumor weight and I) body weight of mice. J) Cell viability analyses were performed in HepG2 and Hep3B cells exposed to sorafenib treated with two pairs of YTHDF2/HSP90β siRNAs or control siRNA for 48 h. K) Co‐IP assay was performed using YTHDF2 antibodies in lysates from HepG2 cells exposed to NVP for 24 h, subjected to the immunoblotting for YTHDF2 and STUB1. *p < 0.05, ^**^ *p* < 0.01, ^***^ *p* < 0.001, ^****^ *p* < 0.0001.

Sorafenib, a multi‐kinase inhibitor, has become one of the most prevalent targeted therapies for advanced HCC. However, the effectiveness of prolonging the overall survival of HCC patients remains limited. Thus, we attempted to determine whether inducing the degradation of YTHDF2 by inhibition of HSP90β may enhance the sensitivity of HCC cells to the targeted therapy with sorafenib. First, we explored the effect of NVP‐AUY922 on the cell proliferation of HCC cells. NVP‐AUY922 notably reduced cell viability and colony formation (**Figure** **6**A,B). Next, we explored the effect of NVP‐AUY922 combined with sorafenib on proliferation in HCC cells. We found that the combination remarkably reduced cell viability and colony formation compared with treatment with NVP‐AUY922 or sorafenib alone (Figure 6C,D). In addition, this combination more obviously induced apoptosis, compared with the alone treatments in HCC cells (Figure 6E).

In order to explore the in vivo effects of the combination, xenograft models were established on nude mice. The results showed that tumor size and tumor weight of HCC xenografts, but not body weight, were remarkably decreased by the combination treatment, i.e., NVP+sorafenib (Figure 6F–I). Additionally, we further confirmed that *HSP90β*‐KD or *YTHDF2*‐KD also restored the sensitivity of both HepG2 and Hep3B cells to sorafenib (Figure 6J). More importantly, inhibition of HSP90β with NVP‐AUY922 increased the interaction of STUB1 and YTHDF2 in HCC cells (Figure 6K), suggesting that NVP targets HSP90β, but not YTHDF2. Together, we demonstrated that the inhibition of HSP90β can enhance the sensitivity of targeted therapy to HCC cells via induction the interaction between STUB1 and YTHDF2.

### 2.7 Clinical Relationship of HSP90β/STUB1 and YTHDF2 in HCC

**Figure 7:**
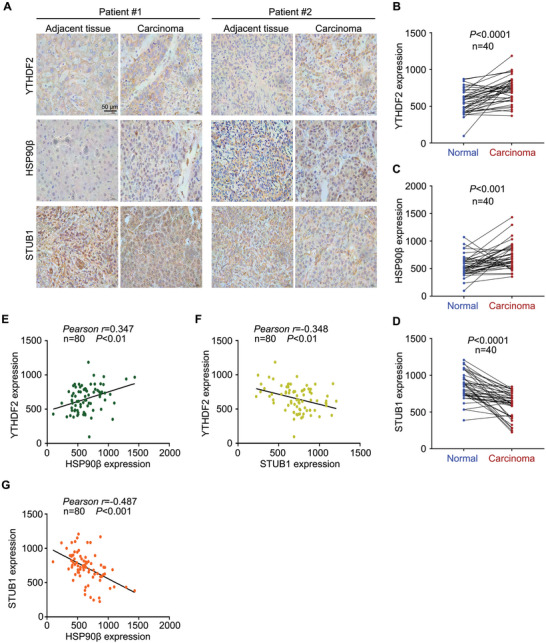
Clinical relationship of YTHDF2, HSP90β, and STUB1 in HCC tissues. A) IHC assay was performed in paraffin‐embedded HCC tissues and adjacent normal tissues using YTHDF2, HSP90β, or STUB1 antibodies. Representative images were shown at 400×. Scale bar, 50 µm. B–D) Quantification of YTHDF2, HSP90β, and STUB1 in (A) was shown. Data were analyzed with paired *t*‐tests. E–G) Correlation analysis of YTHDF2 with HSP90β or STUB1 protein levels, and STUB1 with HSP90β protein levels based on B using Pearson *r* assay. Tumor tissues and adjacent normal tissues were included in the statistics.

**Figure 8:**
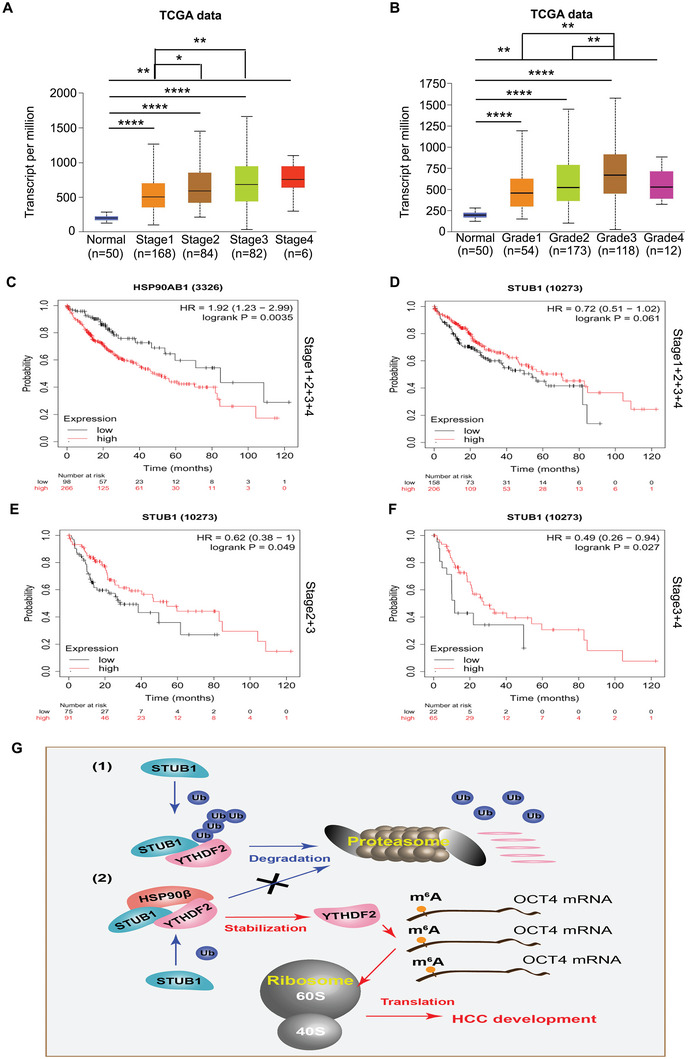
Overall survival analysis of HCC patients using Kaplan–Meier curves. A,B) Analysis of HSP90β mRNA expression in HCC tissues based on cancer stages and tumor grades by analyzing the TCGA and UALCAN databases. *p < 0.05, ^&^ *p* < 0.01, ^&&&^ *p* < 0.0001. C,D) Kaplan–Meier curves from HCC patients expressing low and high HSP90β/STUB1 from the tissue microarray. Overall survival data were shown. E,F) Kaplan–Meier curves from HCC patients (in stage 2+3 or stage 3+4) expressing low and high STUB1 from the tissue microarray. G) A proposed model of HSP90β/STUB1 in the regulation of YTHDF2 in HCC. *p < 0.05, ^**^ *p* < 0.01, ^****^ *p* < 0.0001.

We explored the relationship between HSP90β/STUB1 and YTHDF2 in clinical samples derived from 40 HCC cases to further validate our findings in vitro and in vivo. The immunohistochemistry (IHC) assay showed that protein expressions of YTHDF2 and HSP90β were upregulated, while STUB1 was reduced in HCC tissues, compared with that in normal adjacent tissues (**Figure** **7**A–D). Additionally, protein expression of YTHDF2 was positively correlated with HSP90β expression, while it was negatively correlated with STUB1 expression (Figure 7E,F). Meanwhile, protein expression of STUB1 was negatively correlated with HSP90β expression (Figure 7G). Analysis of the TCGA database via UALCAN showed that HSP90β had higher mRNA levels in various stages or tumor grades of HCC (**Figure** **8**A,B). Moreover, the overall survival analysis with Kaplan–Meier curves showed that higher expression of HSP90β was associated with poor survival in HCC patients, including all stages (Figure 8C). In contrast, higher expression of STUB1 indicated better outcomes in HCC patients, including stage 2–4 (Figure 8D–F). Collectively, our findings on clinical tissues from HCC were highly consistent with the molecular and cellular biology results, further supporting the hypothesis that HSP90β impedes STUB1‐induced ubiquitination of YTHDF2 to drive the growth and sorafenib‐insensitivity of HCC (Figure 8G).

## 3 Discussion

HCC is a challenging and hazardous type of solid tumor. High heterogeneity, drug resistance, postoperative recurrence, and a high risk of metastasis are the leading causes of poor outcomes for patients with HCC. Over the years, sorafenib has been widely used as a first‐line targeted therapy for advanced HCC; ≈30% of patients may benefit from this treatment. However, these patients tend to develop resistance within 6 months.^[^ ^1^, ^3^ ^]^ Therefore, there is still an urgent need to elucidate the molecular and cellular mechanisms of HCC development and progression to excavate more effective intervention measures for the treatment of HCC.

m^6^A is one of the most abundant mRNA modifications. Like many other modifications, m^6^A is also characterized as a dynamic and reversible process. It has been demonstrated that m^6^A regulates the metabolism, maturation, degradation, nuclear output, and mRNA translation, which further regulates various physiological and pathological functions.^[^ ^8^, ^15^ ^]^ YTHDF2, an important reader of m^6^A modification, can selectively bind m^6^A ‐methylated mRNA to control RNA decay in a methylation‐dependent manner.^[^ ^7a^ ^]^ The expression levels of YTHDF2 differ among malignant tumors, and its exact function is still debatable. YTHDF2 is generally upregulated in diverse tumor tissues and has a carcinogenic role.^[^ ^8a^ ^]^ Mechanistically, in prostate cancer, YTHDF2 mediates the mRNA degradation of tumor suppressors, including LHPP and NKX3‐1, to boost AKT phosphorylation‐induced tumor proliferation and migration.^[^ ^16^ ^]^ In addition, YTHDF2 may stabilize the transcripts of MYC and vascular endothelial growth factor A to facilitate tumor progression in glioblastoma stem cells in some potentially indirect manner.^[^ ^17^ ^]^ However, several studies have also been demonstrated its cancer‐suppressing effects in certain models.^[^ ^8^, ^18^ ^]^ This study identified the cancer‐promoting role of YTHDF2 in HCC because the loss of YTHDF2 significantly inhibits tumor growth and sorafenib insensitivity. Our clinical observations showed that YTHDF2 is overexpressed and predicts poor prognosis in patients with HCC.

Previous studies on YTHDF2 mainly focused on its function as an m^6^A binding protein, whereas the molecular mechanisms of how YTHDF2 is regulated at various levels are still unclear. UPS, the selective elimination pathway of proteins to maintain homeostasis, regulates various biological processes. However, the abnormal expression of cancer drivers could result from the dysregulation of protein ubiquitination.^[^ ^4^, ^6^ ^]^ According to the existing reports, the post‐translational modification mechanisms of YTHDF2 include ubiquitination,^[^ ^19^ ^]^ SUMOylation,^[^ ^20^ ^]^ and O‐GlcNAcylation.^[^ ^21^ ^]^ The SUMOylation of YTHDF2 increases its m^6^A modification function and subsequently changes the gene expression profile, thereby promoting the malignant progression of lung cancer.^[^ ^20^ ^]^ In addition, a significant increase in O‐GlcNAcylation of YTHDF2 was observed during hepatitis B virus infection, which may further inhibit its ubiquitination and enhance its protein stability and carcinogenic activity.^[^ ^21^ ^]^ Furthermore, FBW7, a component of the SCF E3‐ubiquitin ligase, may induce ubiquitination of YTHDF2 to suppress ovarian cancer development.^[^ ^19^ ^]^ This study revealed that the ubiquitination level of YTHDF2 was downregulated in tumor tissues of HCC patients compared to normal tissues. Additionally, we identified that the UPS degraded YTHDF2 in HCC cells because inhibition of proteasome with MG132 leads to its ubiquitin accumulation.

In order to further explore the potential mechanism of YTHDF2 regulated by the UPS, we identified the protein interaction between YTHDF2 and molecular chaperone HSP90β using biological mass spectrometry (LC‐MS/MS analysis). According to previous studies, molecular chaperones are involved in regulating ubiquitination and degradation.^[^ ^10^, ^11^, ^12^, ^22^ ^]^ For example, our previous studies have revealed that the molecular chaperone GRP78 binds to the E3 ligase SIAH2 and forms a GRP78‐SIAH2‐AR‐V7 degraded complex to trigger the canonical degradation of AR‐V7.^[^ ^10^ ^]^ Additionally, the mitochondria‐associated molecular chaperone GRP75 recruits the deubiquitinating enzyme USP1 to form a GRP75‐USP1‐SIX1 complex, thereby mediating the deubiquitination and stabilization of SIX1.^[^ ^11^ ^]^ This study identified the protein–protein interactions among HSP90β, YTHDF2, and the E3 ligase STUB1 in HCC cells via co‐IP and exogenous/endogenous IF assays. Moreover, we revealed that the large and small middle domain (276–602 aa) of HSP90β is required for its binding to STUB1 and YTHDF2 in the cytoplasm. At the same time, the N‐terminal (1–384 aa) of YTHDF2 is required for its binding to HSP90β.

Next, the following evidence confirmed that STUB1 promotes ubiquitination and degradation of YTHDF2 in HCC: first, the knockdown of STUB1 did not affect the transcription level of YTHDF2, but prolonged its half‐life, upregulated its protein level, and inhibited its ubiquitination level in HCC cells. In contrast, the overexpression of STUB1 reduced the YTHDF2 protein level, which can be reversed by bortezomib, a proteasome inhibitor. More importantly, we identified that HSP90β acts as a functional inhibitor of STUB1 as it potently suppresses the ubiquitination and degradation of YTHDF2 via binding STUB1 through in vivo and in vitro ubiquitination assays. Further co‐IP analysis revealed that the K245 residue is the critical ubiquitination site on YTHDF2. Functionally, the knockdown of STUB1 promoted cell proliferation, while the knockdown or inhibition of HSP90β significantly limited cancer progression in HCC, similar to the knockdown of YTHDF2. In addition, we revealed that STUB1/HSP90β regulated proliferation in a YTHDF2‐OCT4‐dependent manner. Furthermore, inhibition of HSP90β with NVP‐AUY922 can significantly enhance the sorafenib sensitivity to HCC in both cell lines and xenografts. These findings were consistent with the previous studies that NVP‐AUY922 can attenuate drug resistance in diverse models.^[^ ^23^ ^]^ Moreover, the protein expression and correlation of HSP90β, YTHDF2, and STUB1 were also verified in the clinical samples derived from HCC patients.

Yet, there are several shortages of this study. First, there are multiple cell types especially stroma and noncancerous cells in tumors or normal adjacent tissues. Noises from these nontumorigenic cells cannot be ruled out in the co‐IP assay performed in fresh HCC tissues; second, co‐IP conditions by antibodies are lacking stringency to exclude ubiquitylation signals from YTHDF2 binding proteins as contaminants; third, the effects of sorafenib in combination with NVP‐AUY922 on sorafenib‐resistant model from clinical patients with HCC (such as patient‐derived tumor xenograft) need to be further explored in future.

In summary, this study examined the post‐translational modification of the m^6^A reader, YTHDF2, from the perspective of ubiquitination modification. Our data suggest that HSP90β promotes the growth and sorafenib resistance of HCC cells by suppressing STUB1‐induced YTHDF2 ubiquitination and degradation, which could inaugurate a novel intervention strategy for the clinical treatment of HCC.

## 4 Experimental Section

### Chemicals and Antibodies

Sorafenib (S7397), NVP‐AUY922 (S1069), MG132 (S2619), and cycloheximide (S7418) were obtained from Selleck (TX, USA). Anti‐YTHDF2 (ab246514), anti‐STUB1 (ab134064), anti‐HSP90β (ab203085), anti‐Ubiquitin (ab134953), anti‐K48 Ubiquitin (ab140601), anti‐HA tag (ab9110) were from Abcam (Cambridge, UK). Anti‐Bcl‐2 (15071), anti‐FLAG tag (14793), and anti‐His tag (12698) were from Cell Signaling Technology (Beverly, MA).

### Cell Culture

Embryonic kidney cell line HEK293T and HCC cell lines HepG2/Hep3B were obtained from ATCC. Identities of these cell lines were validated by short tandem repeat profiling. HCC cells were cultured in Roswell Park Memorial Institute (RPMI)‐1640 medium containing 10% fetal bovine serum (FBS), while HEK293T cells were cultured in Dulbecco's modified Eagle medium (DMEM) containing 10% FBS in a humidified atmosphere containing 5%CO_2_/95% air at 37 °C.

### Fresh HCC Samples

The fresh HCC samples, including malignant tumors/adjacent normal tissues, were obtained from the discarded material utilized for routine laboratory tests at the Department of Hepatopancreatic Surgery, First People's Hospital of Foshan (Foshan, China). All procedures were performed with the approval of the Medical Ethics Committee of the First People's Hospital of Foshan (ethics approval number: L[2023] No. 2) and with the full, informed consent of the subjects. The protein extraction steps were performed as previously described.^[^ ^24^ ^]^

### Co‐IP and Immunoblotting Assays

Protein interaction was detected by co‐IP analysis with an Antibody Coupling Kit (Invitrogen). Dynabeads were used to couple the specific antibodies, including STUB1, HSP90β, and YTHDF2, with incubation for 16–24 h. Cell lysates isolated from HCC or HEK293T cells were incubated with Dynabeads‐coupled antibodies. Next, SDS buffer was added to the mixture containing protein‐Dynabeads‐antibodies, followed by incubation at 70 °C for 10 min. Finally, the targeted/combined proteins were isolated from the mixtures via centrifugation. The supernatant was used for further LC‐MS/MS analysis or western blotting, a previously described routine assay.^[^ ^10^ ^]^

### In Vitro Ubiquitination Assay

The in vitro ubiquitination of YTHDF2 was determined using the ubiquitinylation kit (BML‐UW9920‐0001, Enzo Life Sciences, Switzerland) and specific purified proteins. According to the kit instruction, Ubiquitinylation Buffer, E1, E2 (UbcH5a and UbcH5b), Mg‐ATP Solution, Biotinylated Ubiquitin Solution, and human recombinant purified proteins including YTHDF2 (0.5 × 10^−6^ m) (H00051441‐P01, Abnova), STUB1 (100 × 10^−9^ m) (HY‐P71340, MCE), and HSP90β (100 × 10^−9^ m) (ab80033, Abcam) were mixed into a 50 µL ubiquitination reaction system. The reaction mixtures were incubated at 37 °C for 4 h, then boiled with the nonreducing gel loading buffer for 5 min and analyzed by Western blotting.

### LC‐MS/MS Assay

The above co‐IP products were subjected to LC‐MS/MS assay to screen YTHDF2‐interacting proteins. Co‐IP products were first subjected to gel electrophoresis. Next, the protein bands were developed by silver staining, which was further acquired and washed with double distilled water for three times and subjected to decolor reaction. After digestion with trypsin, the samples were centrifugated and dried. Easy nLiquid chromatography (LC) 1200 system (ThermoFisher, USA) was applied to fractionate each tryptic peptide mixture. The trapping, desalting procedure was carried out with a volume of 20 µL 0.1% formic acid. Next, an elution gradient of 80% acetonitrile, 0.1% formic acid was used on an analytical column. Data‐dependent acquisition (DDA) mass spectrum techniques were applied to acquire tandem MS data on a ThermoFisher Q Exactive mass spectrometer (ThermoFisher, USA) fitted with a Nano Flex ion source. Data were acquired using an ion spray voltage of 1.9 kV, and an interface heater temperature of 275 °C. For a full mass spectrometry survey scan, the target value was 3 × 10^6^ and the scan was ranged from 350 to 2000 *m*/*z* at a resolution of 70 000 and a maximum injection time of 100 ms. For the MS2 scan, only spectra with a charge state of 2–5 were selected for fragmentation by higher‐energy collision dissociation with a normalized collision energy of 28. The MS2 spectra were acquired in the ion trap in rapid mode with an AGC target of 8000 and a maximum injection time of 50 ms. Dynamic exclusion was set for 25 s. The MS/MS data were analyzed for protein identification and quantification using A PEAKS Studio 8.5.

### Plasmid/siRNA Transfections

The plasmid (CMV‐MCS‐HA‐SV40‐neomycin) containing full‐length coding DNA sequence (CDS) of human STUB1, plasmids (CMV‐MCS‐3FLAG‐SV40‐neomycin) containing full‐length CDS or various truncated mutants of human HSP90β, and plasmids (CMV enhancer‐MCS‐SV40‐puromycin) containing full‐length CDS or various truncated/point mutants of human YTHDF2 with 6×His at the C‐terminal were generated from GeneChem (Shanghai, China). Plasmids were transfected into HCC cells using RPMI opti‐MEM (Gibco) and lipofectamine 3000 (Invitrogen).

STUB1/YTHDF2/HSP90β/OCT4 siRNAs were purchased from Ribobio (Jiangsu, China). siRNA sequences are listed in Table S1 in the Supporting Information. Briefly, siRNAs were transfected into HCC cells using RPMI opti‐MEM (Gibco) and lipofectamine RNAiMax (Invitrogen) as described before.^[^ ^11^ ^]^

### Lentivirus Transfection

Lentivirus (hU6‐MCS‐Ubiquitin‐firefly_Luciferase‐IRES‐puromycin) containing HSP90β shRNAs (shRNA sequences are listed in Table S2, Supporting Information), lentivirus (Ubi‐MCS‐firefly_Luciferase‐IRES‐Puromycin) containing CDS of HA‐STUB1, and lentivirus (Ubi‐MCS‐SV40‐Cherry‐IRES‐Neomycin) containing CDS of 6×His‐YTHDF2 were purchased from GeneChem (Shanghai, China). For transfection, HCC cells were seeded on a 6‐well plate and cultured to 50% confluence. Supernatant was replaced with medium containing lentiviruses and polybrene (5 µg mL^−1^) at a multiplicity of infection of 10. After incubation for 12 h, supernatant was replaced with medium containing 10% FBS and cultured for 48 h. Puromycin or/and Neomycin were used to select stably transfected cells.

### Reverse Transcription Polymerase Chain Reaction (RT‐PCR) Assay

Total RNAs were isolated from the cultured cells and subjected to real‐time PCR analysis using specific primers for YTHDF2 and STUB1 (sequences listed in Table S3, Supporting Information). This assay was performed with at least three independent repeats, as described before.^[^ ^25^ ^]^

### Immunofluorescence Assay

Cells were seeded in a chamber slide and transfected with HA‐STUB1 plasmids for 48 h. Next, they were washed, fixed, permeabilized, and blocked, as previously reported.^[^ ^26^ ^]^ The primary antibodies anti‐HA tag, anti‐YTHDF2, and anti‐HSP90β were used to bind the specific proteins. Secondary antibodies were used to link the primary antibodies. 4′,6‐diamidino‐2‐phenylindole (DAPI, Abcam, #ab104139) containing resin was used for mounting and nuclear visualization. A confocal microscope (Leica TCS SP8) was used to capture the fluorescent images.

### Proximity Ligation Assay (PLA)

PLA assay was performed using Duolink In Situ Orange Starter Kit Mouse/Rabbit (DUO92102, Sigma‐Aldrich) in HCC cells according to the standard technique. In brief, HCC cells cultured in glass bottom culture dishes were washed with phosphate‐buffered saline (PBS) solution, fixed with paraformaldehyde for 15 min, permeabilized with 0.5% Triton X‐100 for 10 min, and then subjected to blocking for 1 h, primary antibody incubation at 4 °C overnight, Duolink PLA probe (PLUS and MINUS) incubation for 1 h, ligation reaction for 30 min, PCR amplification for 100 min, and finally imaged under a confocal microscope after the final wash by adding Duolink in situ mounting medium containing DAPI. The primary antibodies applied in this assay included anti‐HSP90β (YM0342, Immunoway; ab203085, Abcam), anti‐STUB1 (sc‐133066, Santa Cruz), and anti‐YTHDF2 (ab246514, Abcam).

### Cell Proliferation Assays

Analysis of HCC cell proliferation was assessed by cell viability and clonogenic assays as previously described.^[^ ^27^ ^]^ The MTS Kit (Promega, Peking, China, #G5421) was used for viability assay. After reaching an exponentially growing phase, HCC cells were counted, trypsinized, and 2000–2500 cells per well were plated in a 96‐well plate for 24, 48, 72, 96, and 120 h. After each time point, MTS reagent (20 µL per well) was directly added to each well in dark and incubated for another 2 h at 37 °C. The absorbance at 490 nm was determined using a microplate reader.

For the clonogenic assay, HCC cells were plated in a 6‐well plate (inner diameter, 35 mm) after treatment for the 48 h and cultured for 2 more weeks. After being washed with PBS, the cells were fixed with 4% paraformaldehyde and stained with 1% crystal violet. The images were captured after drying. A diameter >60 µm of the colony under the microscope was included in the analysis.

### IHC Assay

40 cases of paraffin‐embedded HCC and adjacent normal tissues were obtained from the discarded material that was utilized for routine laboratory tests at the Department of Pathology, First People's Hospital of Foshan (Foshan, China). The embedded tissues were sectioned according to standard steps. A MaxVision Kit (Maixin Biol) was used for IHC according to the manufacturer's instruction. The primary antibodies included anti‐YTHDF2, anti‐HSP90β, and anti‐STUB1. All images were captured and quantified as described previously.^[^ ^24^ ^]^

### Animal Study

32 male BALB/c nude mice (5 weeks old) were obtained from Charles River Laboratories (Beijing, China). All the animals were housed in a specific pathogens‐free environment with a temperature of 22 ± 1 °C, a relative humidity of 50 ± 1%, and a light/dark cycle of 12/12 h. All animal studies (including the mice euthanasia procedure) were done after approved by Guangzhou Medical University institutional animal care and use committee (ethics approval number: GY2018‐043), and in compliance with the regulations and guidelines of the committee and conducted according to the ARRIVE guidelines.

For Figure 5, HepG2 cells (5 × 10^6^ cells in 100 µL PBS/mouse) stably expressing 6×His‐YTHDF2 or control plasmids, in the presence or absence of HSP90β shRNAs or HA‐STUB1 plasmids, were subcutaneously inoculated on BALB/c nude mice for 3 weeks (*n* = 8 per group). For Figure 6, mice were randomly divided into four groups (*n* = 8 per group) after subcutaneously inoculating into HepG2 cells (5 × 10^6^ cells in 100 µL PBS/mouse): NVP, sorafenib, NVP+sorafenib, and vehicle group. Mice treated with NVP received i.p. 25 mg kg^−1^/2 days; mice treated with sorafenib received p.o. 20 mg kg^−1^/2 days. An NVP+sorafenib group was first treated with NVP and then with sorafenib. All mice were treated for 3 weeks, after which they were sacrificed by cervical dislocation. Tumor size/weight and body weight were calculated as reported previously.^[^ ^24^, ^28^ ^]^

### Statistical Analysis

Data were presented as mean and standard deviation (SD) from three independent repeats. Paired/unpaired Student's *t*‐tests or one‐way analysis of variance were conducted to determine statistical probabilities where appropriate. SPSS 16.0 and GraphPad Prism 7.0 were used to perform statistical analysis. *p* < 0.05 indicated a statistically significant difference.

## Conflict of Interest

The authors declare no conflict of interest.

## Author Contributions

Y.N.L., Y.L., and C.F.Y. contributed equally to this work. Y.N.L., G.X.C., and H.B.H. conceived the ideas and designed the experiments. Y.N.L., Y.L., C.F.Y., Q.C.L., J.C., W.Y.K., Y.H.Y., X.F.Z., W.S.S., S.S.Y., and G.X.C. performed the experiments. Y.N.L. and H.B.H. wrote the manuscript.

## Supporting information

Supporting Information

## Data Availability

The data that support the findings of this study are available from the corresponding author upon reasonable request.
