# Supplementary material for: HSP90β Impedes STUB1‐Induced Ubiquitination of YTHDF2 to Drive Sorafenib Resistance in Hepatocellular Carcinoma
Source: Adv Sci (Weinh). 2023 Jul 28;10(27):2302025. doi: 10.1002/advs.202302025 (PMC10520652; doi:10.1002/advs.202302025)
Supplement: Supplementary file 1 — Supporting Information [file ADVS-10-2302025-s001.pdf]

ADVANCED SCIENCE
Open Access

Supporting Information

for *Adv. Sci.*, DOI 10.1002/advs.202302025

HSP90*β* Impedes STUB1-Induced Ubiquitination of YTHDF2 to Drive Sorafenib Resistance in Hepatocellular Carcinoma

Yuning Liao, Yuan Liu, Cuifu Yu, Qiucheng Lei, Ji Cheng, Weiyao Kong, Yuanhui Yu, Xuefen Zhuang, Wenshuang Sun, Shusha Yin, Gengxi Cai* and Hongbiao Huang**

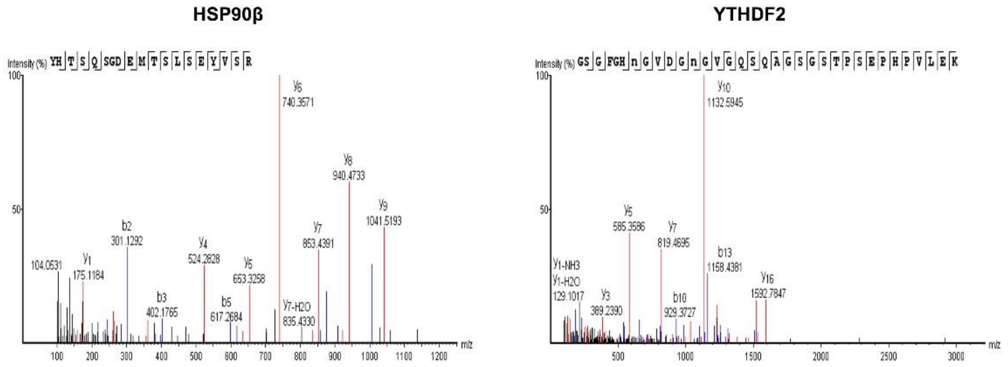

Figure S1, related to Figure 2. Mass spectrograms of YTHDF2 and HSP90β.

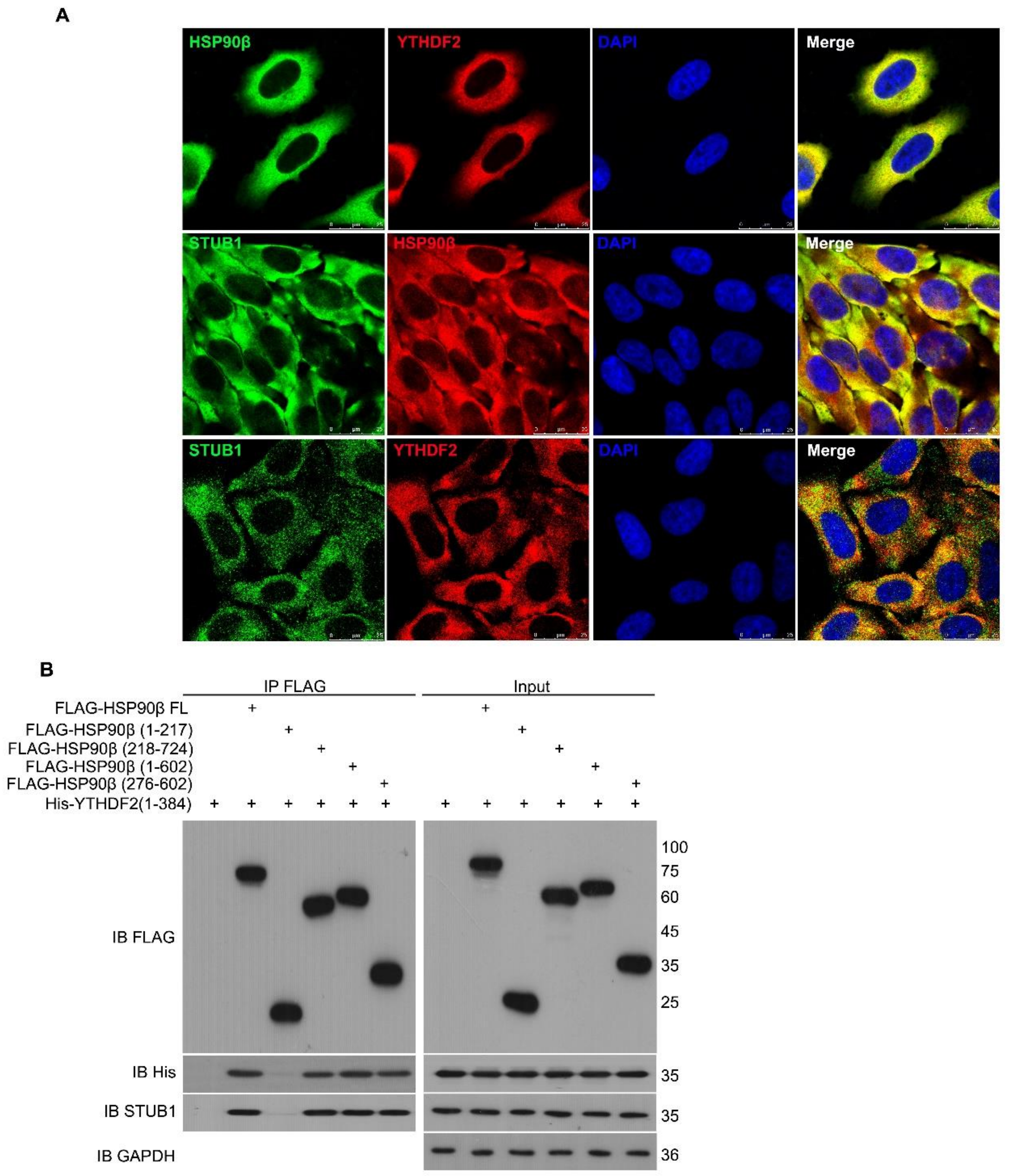

Figure S2, related to Figure 2. Protein interaction among HSP90β, STUB1, and YTHDF2. (A) Endogenous immunofluorescence assay was performed in HepG2 cells using anti-STUB1, anti-YTHDF2, and anti-HSP90β. Scale bars, 25 μm. **(B)** Truncated mutants of FLAG-HSP90β plasmids were transfected with 6×His-YTHDF2 (1-384 aa) plasmids into HepG2 cells for 48 h. Co-IP assay was performed using anti-FLAG, followed by immunoblotting for FLAG, His, and STUB1.

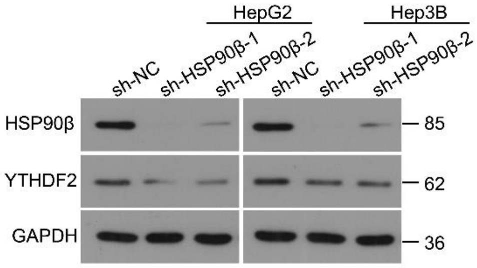

Figure S3, related to Figure 3. HSP90β increases the expression of YTHDF2. Immunoblot for HSP90β and YTHDF2 was performed in HepG2 and Hep3B cells stably expressing HSP90β shRNAs or control shRNA.

Table S1. The target sequences of siRNAs used in the study.

siRNA	Target sequence
YTHDF2 siRNA-1	5'-GCACAGAAGTTGCAAGCAA-3'
YTHDF2 siRNA-2	5'-GGTAGCGGGTCCATTACTA-3'
STUB1 siRNA-1	5'-GGAGATGGAGAGCTATGAT-3'
STUB1 siRNA-2	5'-GGAGCAGGGCAATCGTCTG-3'
STUB1 siRNA-3	5'-CTGTGAAGGCGCACTTCTT-3'
HSP90β siRNA-1	5'-AAGTGGTTGTGATCACAAA-3'
HSP90β siRNA-2	5'-CGACAAGAATGATAAGGCA-3'
OCT4 siRNA-1	5'-GTATTCAGCCAAACGACCA-3'
OCT4 siRNA-2	5'-TATTCAGCCAAACGACCAT-3'

Table S2. The target sequences of shRNAs used in the study.

shRNA	Target sequence
HSP90β shRNA-1	5'-GAAGTTGGACAGTGGTAAA-3'
HSP90β shRNA-2	5'-AAGTGGTTGTGATCACAAA-3'

Table S3. The target sequences of primers used in the study.

Primer	Target sequence
GAPDH (Forward primer)	5'-ATCATCCCTGCCTCTACTGG-3'
GAPDH (Reverse primer)	5'-GTCAGGTCCACCACTGACAC-3'
YTHDF2 (Forward primer)	5'-AGCCCCACTTCCTACCAGATG-3'
YTHDF2 (Reverse primer)	5'-TGAGAACTGTTATTTCCCCATGC-3'
STUB1 (Forward primer)	5'-AGGCCAAGCACGACAAGTACAT-3'
STUB1 (Reverse primer)	5'-CTGATCTTGCCACACAGGTAGT-3'
